# A happy cell stays home: When metabolic stress creates epigenetic advantages in the tumor microenvironment

**DOI:** 10.3389/fonc.2022.962928

**Published:** 2022-08-26

**Authors:** Eric A. Hanse, Mei Kong

**Affiliations:** Department of Molecular Biology and Biochemistry, University of California, Irvine, Irvine, CA, United States

**Keywords:** tumor, metabolism, glutamine, epigenetics, alpha ketoglutarate, glutaminolysis-inhibition, tumor microenvironment

## Abstract

A paradox of fast-proliferating tumor cells is that they deplete extracellular nutrients that often results in a nutrient poor microenvironment *in vivo*. Having a better understanding of the adaptation mechanisms cells exhibit in response to metabolic stress will open new therapeutic windows targeting the tumor’s extreme nutrient microenvironment. Glutamine is one of the most depleted amino acids in the tumor core and here, we provide insight into how important glutamine and its downstream by-product, α-ketoglutarate (αKG), are to communicating information about the nutrient environment. This communication is key in the cell’s ability to foster adaptation. We highlight the epigenetic changes brought on when αKG concentrations are altered in cancer and discuss how depriving cells of glutamine may lead to cancer cell de-differentiation and the ability to grow and thrive in foreign environments. When we starve cells, they adapt to survive. Those survival “skills” allow them to go out looking for other places to live and metastasize. We further examine current challenges to modelling the metabolic tumor microenvironment in the laboratory and discuss strategies that consider current findings to target the tumor’s poor nutrient microenvironment.

## Introduction

One of the most significant movements of the last ten years in cancer research has been toward efforts aimed at therapeutically targeting cancer metabolism. As we have learned, cancer cells dysregulate carbon circuits in surprising ways to fulfill their needs. At first glance, the nutritional requirements of the proliferating cell seem obvious. They need to double their biomass to generate copies of themselves, this requires an increased demand for, and access to lipids, nucleic acids, amino acids and carbohydrates for building blocks (1–3). Not surprisingly, many of these reactions are working against concentration gradients and must overcome high energy thresholds to provide the tumor cell with what it needs to survive (1, 4). Cancer cells have showed clever ways to overcome supply and demand issues. And more and more we are identifying nutrients that cancer cells are dependent upon.

One of the more important leaps made by translational scientists during this time was the idea that deprivation of these nutrients was the key to killing cancer. This has been and will continue to be a noble endeavor. Choking out the cell from what it needs is logical. Moreover, the metabolic demands of cancer cells are usually different than the demands of healthy and/or quiescent cells making these highly desirable intervention points for therapies. However, we argue here that caution must be taken using nutrient deprivation as a strategy considering emerging data that suggest metabolic adaptations could drive durable resistance in tumors. These recent studies should be considered as we try to translate the cancer cell metabolism learned over the past century into viable treatments for humans.

## Glutamine ties metabolism to epigenetics through αKG

Glutamine and glutaminolysis have been in the crosshairs of many cancer labs over the past decade. Glutamine provided a very important piece of the puzzle Otto Warburg had left us with in the early 1900’s (5). If glycolysis was ramped up to produce lactate, Warburg and others had postulated that the mitochondria of cancer cells were defective. As technology developed, however, it was found that in fact mitochondria were fully operational and in some cases more active in cancer cells (6). Enter glutamine, who in two steps could provide mitochondria with the αKG required to keep the TCA cycle turning with little to no carbon from glycolysis (7–9). The glutaminolysis enzymes have even been found to be tightly controlled by oncogenes such as Myc (10). Since its initial discovery, there has been much work done showing dependence of glutamine by cancer cells (8, 11–17). This has led to momentum toward drugs entering clinical trials that target glutaminase and hold great clinical promise (18).

**Figure 1:**
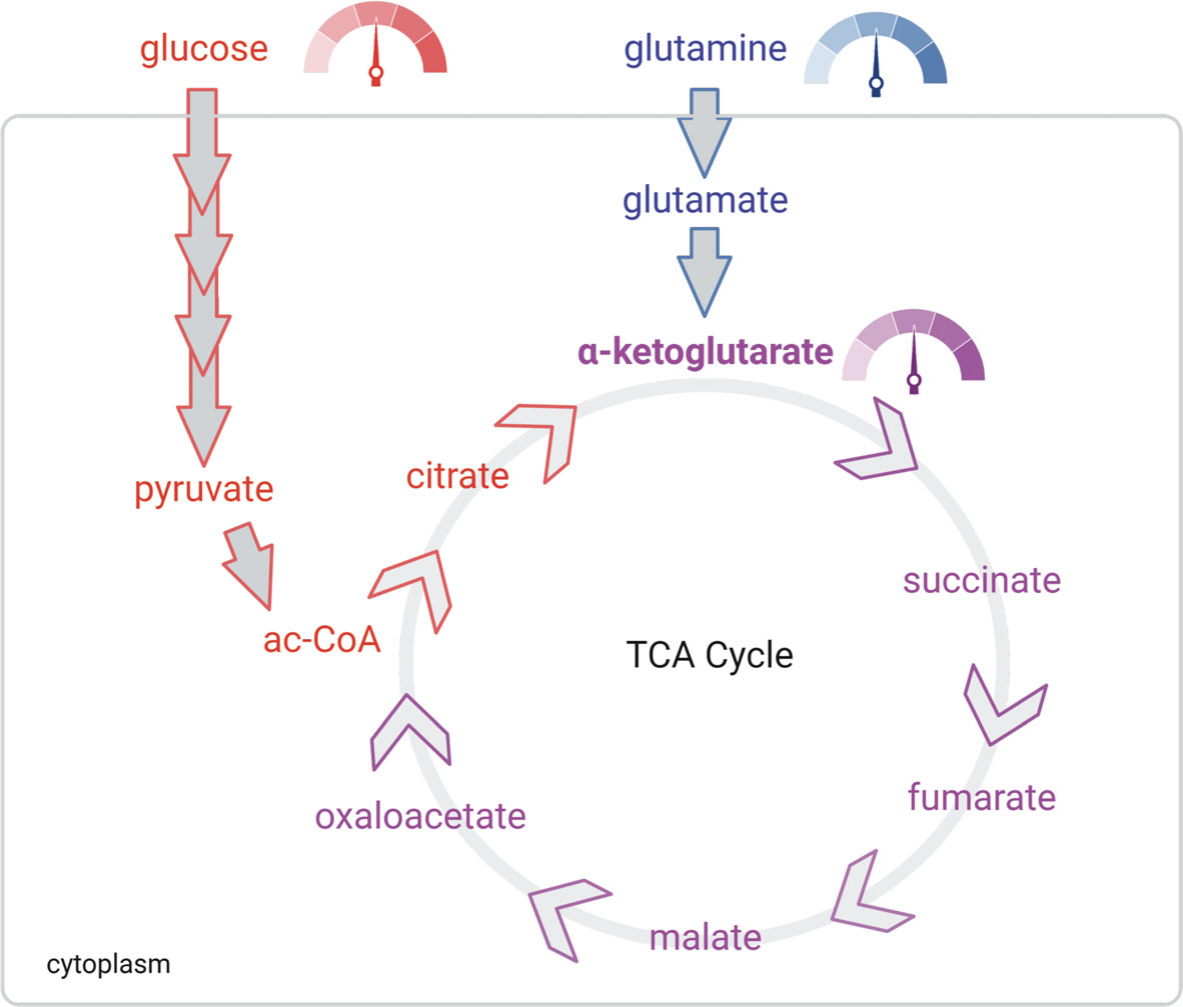
αKG is a gauge of extracellular nutrient supply. Glucose (red) and glutamine (blue) supply lines meet at αKG (purple). The TCA is controlled mostly by the demand of the electron transport chain. Thus, αKG concentrations signal to the cell the energy producing potential taking inputs from both supply (extracellular concentrations of glucose and glutamine) and demand (TCA) of the cell.

**Figure 2:**
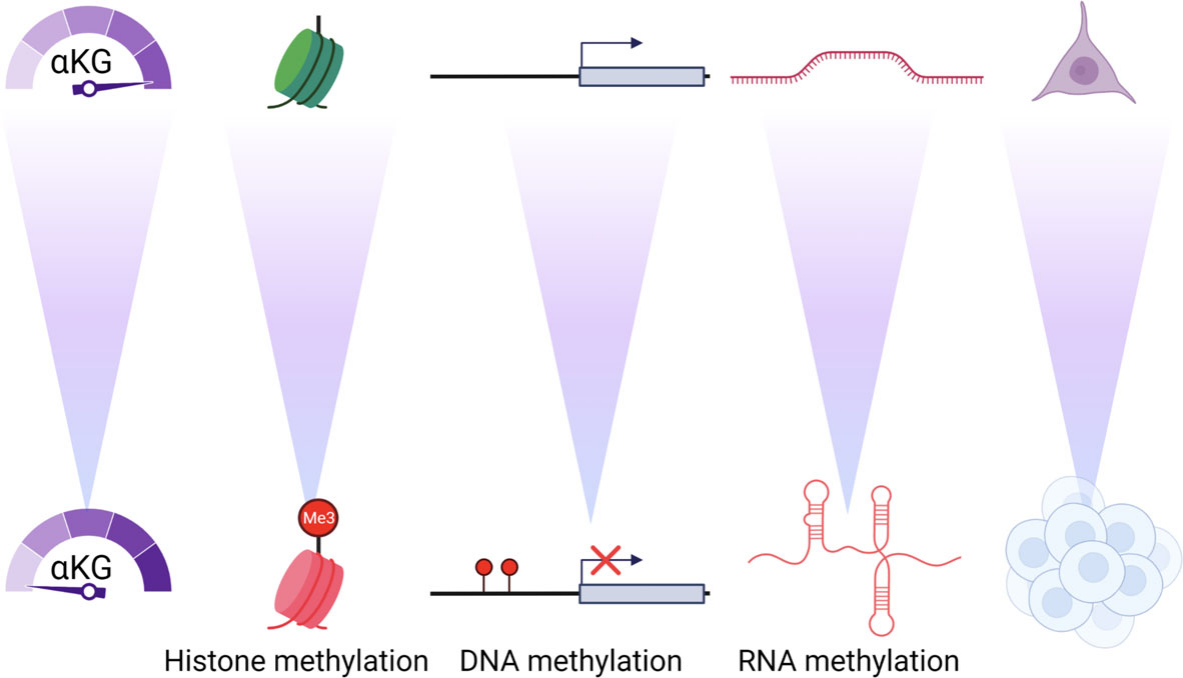
αKG concentrations control the epigenome and differentiation. As αKG concentrations decrease there is an increase in the methylation of histones, DNA and RNA that causes profound and durable changes to the differentiation and phenotype of the cell.

**Figure 3:**
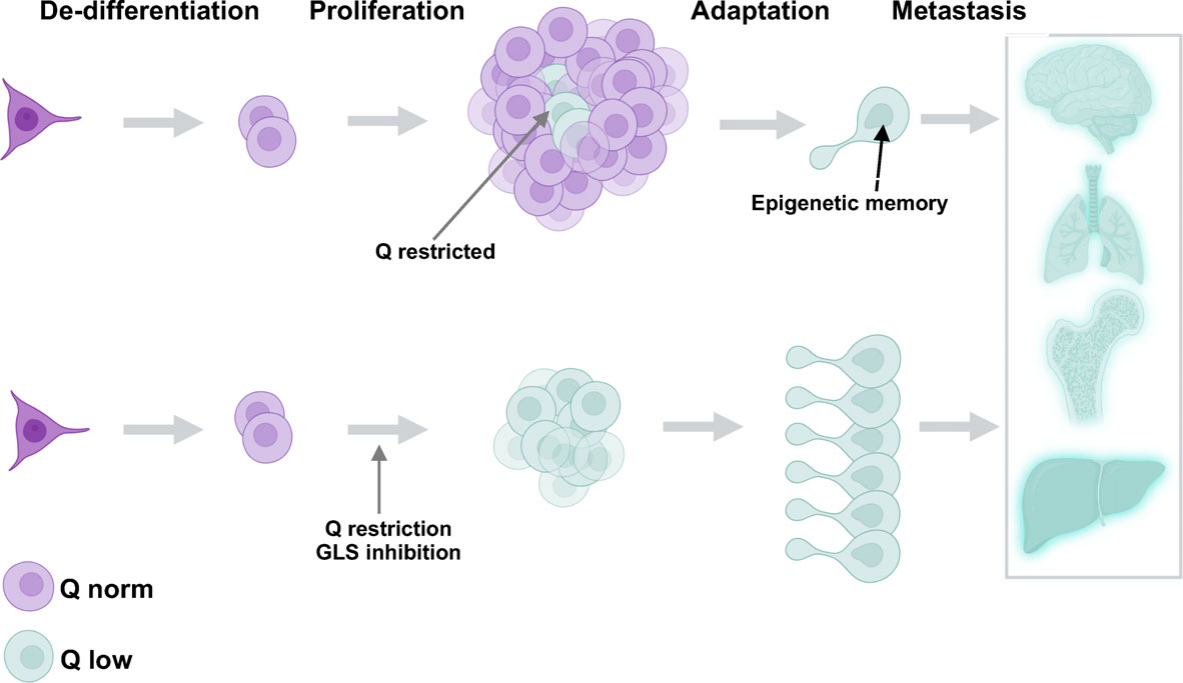
Glutamine restriction has the potential to generate more cells with metastatic potential through epigenetic adaptation. Glutamine restricted cells (aquamarine) are naturally formed in the tumor center. These cells become epigenetically transformed and exhibit the potential to metastasize and invade other tissues (upper panel). Glutamine restriction is potentially a tumor de-bulking strategy (lower panel) but may give rise to many more adapted and dangerous cells capable of metastasis.

As glutamine’s rise to prominence became more apparent, other significant metabolic pathways have emerged that play a role in cancer progression. Some of these roles have even been broadened beyond carbon shuttling. One in particular that has shown importance in influencing phenotypes is α-ketoglutarate (αKG) (**Figure 1**). In general, αKG is a co-factor for demethylase enzymes that remove methyl groups from target substrates (19, 20). The dioxgenase activity of the enzyme requires αKG for the hydrolysis reaction. As for DNA demethylases, αKG acts on the Ten eleven translocation (Tet) genes that remove methyl groups from the cytosine-guanine rich regions located at or near the promoters of genes. The result of decreased or competitive inhibition with αKG in these cells is an increased methylated genome (20). For DNA packaging, histone demethylation is required to allow access to transcriptional machinery. αKG is co-factor for the Jumonji domain-containing histone demethylases (Jmjc). These de-methylases also play a major role in cancer and decreased activity of these enzymes, through direct competition with αKG is observed in gliomas and other tumor types (19, 21). The ability of glutamine to generate αKG and thus influence the enzymatic activity of DNA, RNA and histone demethylases is a potential major factor in tumor evolution we have not fully realized (**Figure 2**). It seems evolution has developed an αKG dependent method to monitor environmental access to carbon and bring about global phenotypic changes. Some of these pathways may be left over from development when it was shown that TCA metabolites can alter the differentiation fate of cells (22–25). Indeed, it is likely these metabolic conditions are limited to the unique microenvironment during development. Interestingly, a recent study has tied glucose and glutamine together at an intersection that drives lipid metabolism, another critical metabolic adaptation in cancer cells (26). These findings are another important example of mechanisms that have evolved to sense and adapt to carbon levels. Alpha-ketoglutarate is at an interesting metabolic intersection where glycolysis and glutaminolysis combine (**Figure 1**). As such, evolution may have carved out a role for αKG to serve as a general signal of the metabolic environment to the cell and many reports have demonstrated how overcoming low glutamine concentration and thus αKG signaling are critical responses for tumorigenesis (19, 20, 27–31). For example, melanoma cells grown in low glutamine medium metastatically colonize to the lung more readily (32). And, in a model of colorectal cancer, intestinal organoids cultured in low glutamine are less differentiated and more invasive (29). Hypoxia, another stress experienced at the tumor core, has similar effects on driving the invasiveness and metastatic nature of the tumor (33–35). Interestingly, it stands to reason that glutamine and oxygen availability in the tumor are mostly supplied by blood meaning cells experience low oxygen in tandem with low glutamine. Taken together, it is becoming clearer that nutrients, and in particular glutamine, have a major role to play in the adaptation and survival of nutrient stressed cancer cells (**Figure 3**).

## Restoring cellular αKG levels blocks epigenetic adaptation

When the ability to control αKG dependent enzymes is lost, tumors tend to become more advanced. Nowhere is this more illustrative than in gliomas that harbor mutations in the isocitrate dehydrogenase enzyme which produces the αKG analog 2-hydroxyglutarate. This metabolite, the result of a defective enzyme, accumulates in the cell and outcompetes αKG for access to the enzyme (20). In fact, 2-HG on its own is sufficient to induce a tumor phenotype in non-transformed cells making it an oncometabolite (27). The implication from these studies is that αKG in high concentrations could prove to have opposite effects of the oncometabolite 2-HG and be tumor suppressive. We and others have reported αKG treatment results in a more differentiated phenotype (22–24, 29, 36–39). In colorectal cancer, we found αKG treatment had global effects on the epigenome along with repressed Wnt signaling and differentiation phenotypes (29). Cancer cells which are undoubtedly glutamine hungry are thus faced with a problem of increased DNA and histone de-methylation. On the flip side, a growing solid tumor living beyond its blood supply line may be faced with nutrient starvation that limits access to glutamine and αKG. Thus, a balance must be struck for the tumor to survive and thrive. One major concern is that broad changes to the epigenome are selecting for cells that can survive harsh environments, and environments they were not intended to live in, giving rise to cells that harbor more metastatic potential.

The decreased concentration of glutamine and thus αKG at the tumor core could be a barrier to durable therapies. One way to address this challenge would be to increase nutrient levels in the blood to prevent some of these adaptations and there is some evidence these approaches could be useful. For example, in melanoma, a more differentiated tumor is observed simply by increasing glutamine in the diet (40). These studies reveal the same mechanisms modulating the epigenome as was observed in CRC; the increased αKG concentrations as a result of the high glutamine diet were successful at modulating the epigenome toward more differentiated tissue (29). Importantly, this study suggests modulating αKG intracellularly could be as easy as increasing glutamine supplementation in the diet or water. It seems an increased dependence on one-carbon metabolism could propose a potential in route to making αKG treatments more effective. For example, methionine cycling pathways are often accelerated in cancers (41). A consistently high thirst for glutamine can lead to increased αKG concentrations which in turn signal to the epigenome through de-methylation. The counter to this seems to be re-methylation and thus a dependence on methyl substrates. Indeed, several elegant studies have described the importance of one carbon metabolism (OCM) in cancer and how disabling these pathways could be beneficial (42–45). Luckily, these re-methylation adaptations in glutamine hungry cells could be in tandem with OCM drugs that are already used in the clinic. Interestingly, and in line with the idea that overcoming αKG repression is critical to tumor development, OCM inhibitors have been used in the clinic successfully for decades. Methotrexate and decitabine are currently clinically applied for the treatment of a variety of cancers from leukemia to sarcoma. It seems the chronic demethylation catalyzed by the αKG concentration is compensated for by an enhanced re-methylation program. Thus, keeping the cell from re-methylating the oncogenic epigenome and DNA methylome precipitated by high levels of αKG in glutamine hungry cells.

## Epigenetic adaptations are remembered in cancer cells

In terms of biological timeline, cancer biologists have understood phenotypic changes in response to environmental stress as either immediate-early in terms of transcriptional regulation or longitudinally with the acquired mutations that drive tumor evolution. This is where epigenetics has become the forerunner in driving “midterm” adaptations that allow the cell to survive in harsh environments, and sometimes unintentionally in the environments of other tissues as other metastatic disease. The cell’s ability to adapt to potential long term nutrient deprivation can be controlled on a global genomic scope by epigenetics. Histone marks and DNA methylation control access of transcriptional machinery to the gene and thus have a rheostat like control on global transcription. Recently, RNA epigenetics have been implicated in cancer (**Figure 2**) (46, 47). These modifications are methylation marks that change the structure and function of the molecules and are found on all species of RNA. However, it is thought long non-coding and miRNAs are impacted the most by epigenetics by adding more adaptability and inputs to the regulatory potential of these RNAs. Indeed, two of the main RNA de-methylases ALKBH5 and TET are αKG dependent dioxgenase enzymes suggesting glutamine depravation will also affect the epitranscriptome of cancer cells (20, 48). We are just beginning to understand the implication of these marks but based on these studies can infer that RNA epigenetics will also be dependent upon αKG levels. Indeed, the global epigenetic changes observed in human cancer are thought of as necessary adaptations that result in silencing critical tumor suppressors (49). In gliomas, where 2-HG out-competes αKG for access to demethylases, a global epigenetic shift is observed in the tumor (50). Interestingly, global DNA methylation is increased in colorectal tumors compared to normal tissue (51). And importantly colorectal cancer has been shown to be responsive to changes in diet both positively and negatively (52–54). It’s tempting to speculate diet interventions that target de-methylation such as those of high glutamine in CRC tumors could hold promise for successfully blocking tumor progression. Along those lines, influencing the epigenome and epitranscriptome could also be avenues to treating tumors that result in more durable responses for CRC in the clinic. Preserving epigenetic memory in cancer is an important adaptability mechanism that prepares the cell to live in metabolically harsh environments. As such we propose a low glutamine microenvironment not only produces cells with high methylation epigenetics, but will also produce cells with durable epigenetic adaptation for tumor fitness, increasing the danger of the tumor burden.

## Deprivation is different than depleted

**Figure 4:**
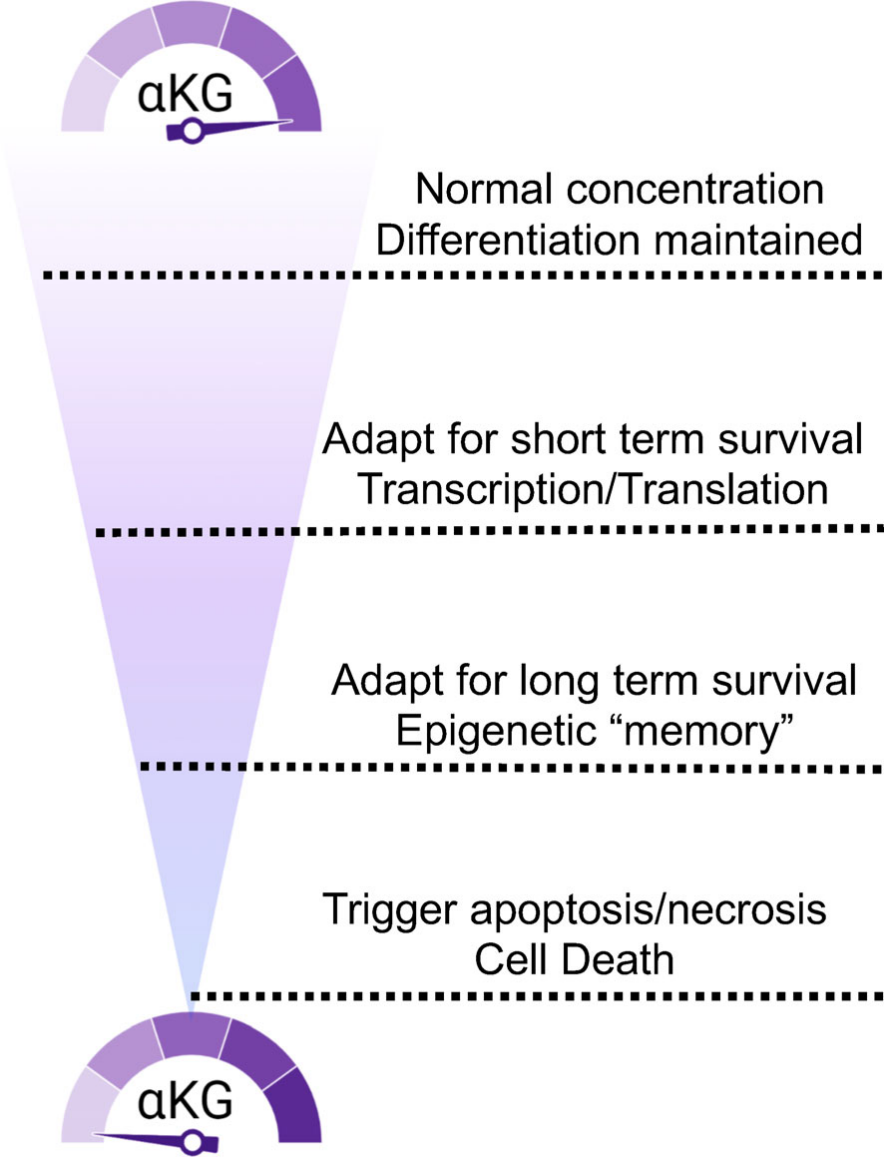
αKG concentration fluctuations trigger a spectrum of cellular responses. Access to glutamine and αKG trigger responses that range from immediate need to epigenetic durability to death.

The golden age of metabolism research started 20 years ago, with scientists trying to piece together metabolic pathways with significant roles in supporting tumor development. Nevertheless, many of these experiments were performed in established media that were formulated as richly as possible to get cells to grow outside of the body. Metabolism, out of necessity is a reductionist discipline. Rigorous efforts have shown us over decades how cancer cells respond to nutrient stress. Just as with drugs that target proliferation, the challenge remains how to distinguish between healthy and cancer. We now appreciate that cells adapt to nutrient stress on a spectrum. Cells must monitor stress to navigate the subtle and sometimes harsh *in vivo* environment. It seems cellular metabolism is altered in response to the degree of this stress and not with a binary switch as we first believed. Moving forward, we need to revisit a spectrum of adaptations and make sure we are targeting a metabolic version of the cell that is not preparing for escape. In the future, models that better mimic that tumor microenvironment to provide physiological levels of nutrients will be critical tools to bring about new and exciting directions to the cancer metabolism field. Thus, increasing the focus on the adaptive mechanisms that happen when nutrients are limited but not gone may open up new avenues of investigation and therapeutic targeting. The aggregation of this knowledge has led us to propose a simplified model of glutamine/αKG metabolic adaptation when considering the response to glutamine depravation. Moreover, a variable missing from our model and not yet thoroughly investigated by the field is time. Of course, not only the longevity of nutrient challenges but also the cyclical nature of biology continue to be unknowns (**Figure 4**).

## The tumor microenvironment is still a metabolic mystery

Metabolism as a discipline made a resurgence in the 2010s when technologies such as Seahorse, isotopomer tracing and mass spectrometry became more accessible. We became very good at predicting how cancer cells would metabolize basic building block nutrients in culture. However, when progressive leaps into *in vivo* studies began to emerge, we found that metabolism in a complex environment is much different than the phenotypes we encounter *in vitro*. For example, a number of groups have found and described how critical scavenging from the microenvironment is to tumor metabolism, generally revealing a more aggressive and resistant tumor (25, 26, 55–57). The reductionist nature of basic metabolism has given us a great foundation to stand on. We know what the cancer cells want and what they do with it. However, these observations have mostly been made in a controlled environment. Inside the body, not surprisingly, the tumor cells are encountering a biological milieu we are only beginning to understand.

The microenvironment on its own is a hot topic of study and we have understood how the crosstalk between tissues and cells influence the development of tumors. Some of the most efficacious cancer drugs available are extracellular antibodies that interfere with cross talk in the tumor microenvironment by either blocking cell to cell contact or hormonal/receptor interference. Metabolically, however, we are only beginning to scratch the surface of how metabolites from outside cells are influencing the tumor. For example, Elia et al. found that exogenous pyruvate increases the EMT capacity of breast cancer cells (57). The current therapeutic focus on tumor immunology has shown us nuanced metabolic relationships between lymphocytes and the tumor in the microenvironment affects the ability of immunosurveillance to clear tumors (58, 59) Considering the power of how metabolites are not simply building blocks they also have epigenetic altering power, efforts investigating the metabolic microenvironment will help us get to therapies that compensate for the reprogramming and adaptations that happen both intrinsically and extrinsically *in vivo*.

## A happy cell stays home, therapeutic strategies moving forward

What happens when we limit the supply of oxygen and nutrients to a cancer cell? A significant portion of the tumor will succumb to the starvation and the bulk tumor will decrease. However, these extreme environments also cause cells to adapt with mutation, to remember with epigenetics and to emerge as a cell with the ability to grow in restricted environments. It will be challenging to approach tumors with the idea that starvation or nutrient deprivation will have deleterious effects for patients. Instead, we argue that the low nutritional microenvironment triggers epigenetic changes that not only adapt the cells to better survival skills *in situ*, but also rewire terminal differentiation and enhance metastatic potential. The latter being one of the major problems with resistance and recurrence seen in patients. We and others have described the transcriptome and phenotype in response to glutamine deprivation can be avoided by shutting down the epigenetic modifications brought on by low αKG and demethylation of the epigenome (29, 40). One strategy would be to inhibit the re-methylation circuits up regulated by glutamine deprived cells or inhibit the one carbon metabolism pathway, which on its own is shown to be enhanced in tumors (43–45). The parallel loss of essential building blocks while inhibiting its adaptive maneuvers will ensure a better outcome and avoid the resistant epigenetic signatures that drive EMT and metastasis.

**Figure 5:**
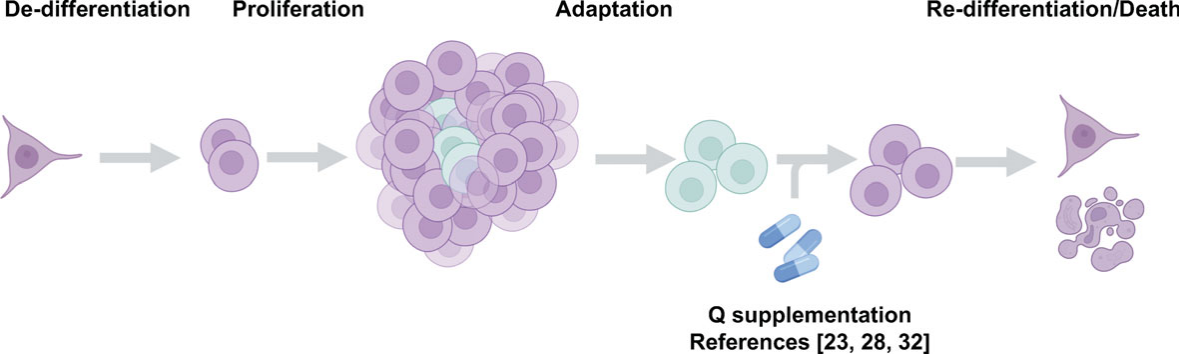
Glutamine supplementation reverses the de-differentiated phenotype of cancer cells. Recent studies from our lab suggest glutamine supplementation in melanoma (37, 40) and colorectal cancer (29) is a potential strategy to limit the formation of the dangerous de-differentiated cells found in the tumor core.

**Figure 6:**
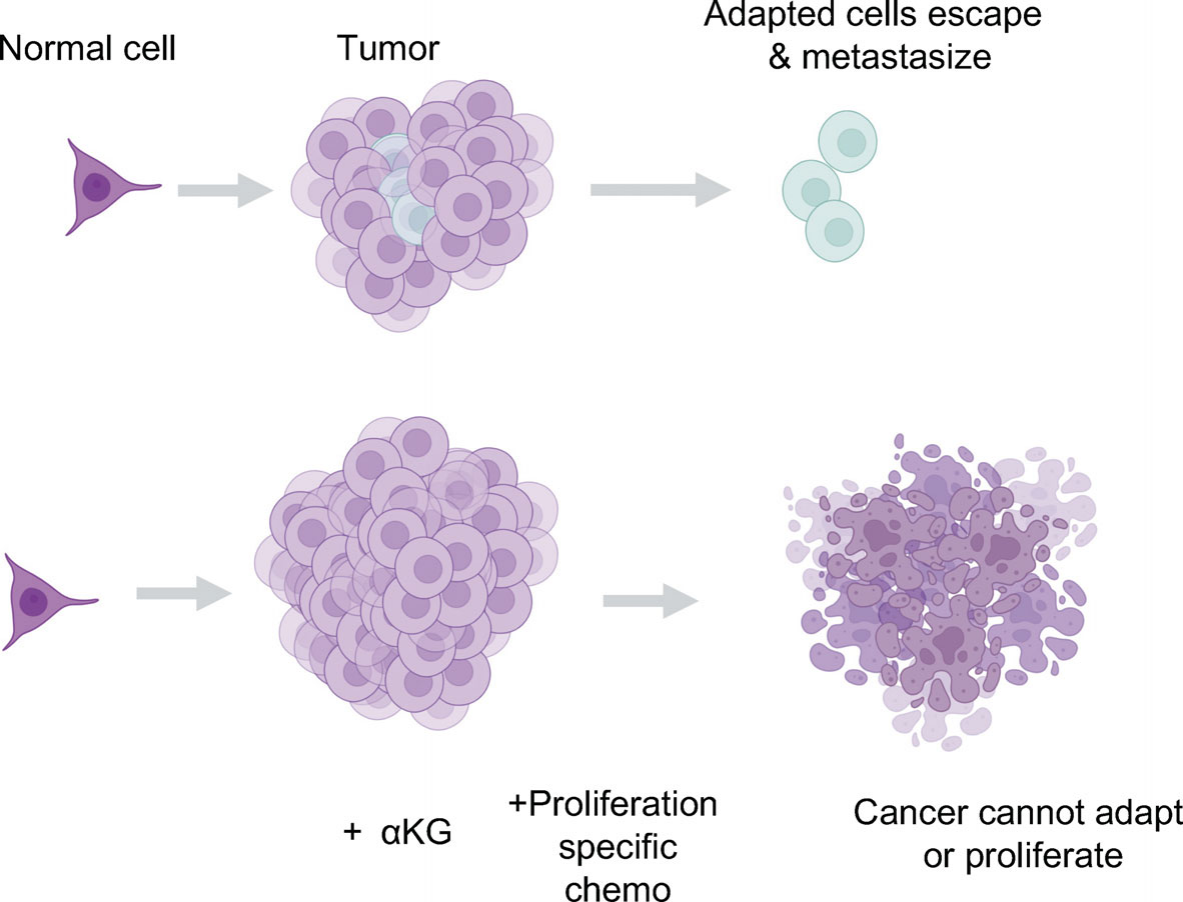
The potential for αKG to synergize with standards of care as a therapeutic. We propose that supplying the breakdown of the glutamine supply line is a potentially dangerous therapeutic strategy. Instead, we propose a combination of proliferative targeting drugs currently available that do not allow tumor growth, in tandem with glutamine or αKG supplementation to maintain the differentiated and metabolically maladapted state of the cell limiting its ability to survive in a tumor or more importantly, outside of the tumor and into other tissues.

As eluded to above, drug resistance has been and continues to be a barrier to therapeutic progression. Therapies that have targeted both proliferation and supply lines have been traditionally susceptible to metastatic recurrence. A happy cell stays home, whereas a starving cell will look for food, acquire new tastes and even change identities to survive. Current therapies may be unintentionally driving more aggressive tumors by limiting nutrients such as glutamine (**Figure 4**). In addition, the systemic inhibition of glutamine metabolism is likely to be an obstacle to the effectiveness of other tissues. For example, recent findings revealed that glutaminase inhibition surprisingly blocked the T cell response to lung tumors (60). Beyond the immune system glutamine and glutamine metabolism are critical to many tissues like brain, liver and muscle. So, how can we limit the biomass the tumor needs to survive without compromising the adaptive response? Recent data from our lab suggests glutamine supplementation can reverse a de-differentiated phenotype of cancer cells (**Figure 5**). The solution in our eyes, is to limit metabolic stress on the tumor so as to inhibit epigenetic adaptation. We need to focus on combinatorial methods for these promising metabolic inhibitors. Many of the experiments testing single agents have had difficulty translating into the clinic or getting momentum in the pharmaceutical world. We understand, more than ever the complexity of the tumor also requires complex therapeutic design. The drugs this field has generated are exquisitely effective and still hold tremendous promise. The burden will be on us to test these agents using the knowledge gained and highlighted in this review. Here, we propose that a combination of nutrient supplements with traditional therapeutics that target general proliferation are the most obvious to start. A major criticism to this approach is the idea that we would be feeding the tumor, providing it with the biomass it wants to grow more rapidly. However, with the proliferation drugs on board, we feel that the benefits of epigenetic maintenance provided by stable nutrient supplies far outweighs the resistance driving nature of starvation tactics (**Figure 6**). Since proliferation drugs are well established in the clinic and effective, we already have a tool to control tumor outgrowth in hand. Moving forward, it will also be interesting to test glutamine restriction with drugs that de-stabilize the epigenome and thus dis-allow the stem cell transition that happens as a result of glutamine restriction. Another option would be to combine glutamine restriction with drugs that target or limit one carbon metabolism in order to disrupt the methylation cycle compensated for by the loss of αKG. Moreover, these approaches, combined with early detection and genetic predisposition screening could yield a new direction for cancer therapy that involve more nutrient supplementation and less chemical intervention. Learning to control a tumor rather than remove it could be achievable given the knowledge we have gathered to this point and the directions technology are taking us.

## Author contributions

EH and MK conceived and wrote the manuscript. All authors contributed to the article and approved the submitted version.

## Funding

EH is supported by American Cancer Society PF DDC-132846. MK is supported by National Institute of Health R01CA244360 and R01GM132142.

## Acknowledgments

We would like to thank members of the Kong lab for productive and helpful discussions.

## Conflict of interest

The authors declare that the research was conducted in the absence of any commercial or financial relationships that could be construed as a potential conflict of interest.

## Publisher’s note

All claims expressed in this article are solely those of the authors and do not necessarily represent those of their affiliated organizations, or those of the publisher, the editors and the reviewers. Any product that may be evaluated in this article, or claim that may be made by its manufacturer, is not guaranteed or endorsed by the publisher.
